# Pressure-Sensitive Capability of AgNPs Self-Sensing Cementitious Sensors

**DOI:** 10.3390/s23249629

**Published:** 2023-12-05

**Authors:** Haoran Zhu, Min Sun

**Affiliations:** School of Civil Engineering, Suzhou University of Science and Technology, Suzhou 215011, China; 18862161767@163.com

**Keywords:** self-sensing cementitious, silver nanoparticles, stress-sensitive, dispersion, long-cycle loading test

## Abstract

Intelligent monitoring approaches for long-term, real-time digitalization in structural health monitoring (SHM) are currently attracting significant interest. Among these, self-sensing cementitious composites stand out due to their easy preparation, cost-effectiveness, and excellent compatibility with concrete structures. However, the current research faces challenges, such as excessive conductive filler, difficulties in filler dispersion, and insufficient stress sensitivity and instability. This study presents a novel approach to these challenges by fabricating self-sensing cementitious sensors using silver nanoparticles (AgNPs), a new type of conductive filler. The percolation threshold of AgNPs in these materials was determined to be 0.0066 wt%, marking a reduction of approximately 90% compared to traditional conductive fillers. Moreover, the absorbance test with a UV spectrophotometer showed that AgNPs were well dispersed in an aqueous solution, which is beneficial for the construction of conductive pathways. Through various cyclic loading tests, it was observed that the self-sensing cementitious sensors with AgNPs exhibited robust pressure-sensitive stability. Additionally, their stress sensitivity reached 11.736, a value significantly surpassing that of conventional fillers. Regarding the conductive mechanism, when encountering the intricate environment within the cementitious material, AgNPs can establish numerous conductive pathways, ensuring a stable response to stress due to their ample quantity. This study provides a significant contribution to addressing the existing challenges in self-sensing cementitious materials and offers a novel reference for further research in this domain.

## 1. Introduction

Structural health monitoring (SHM) is a vital research area in civil engineering, aimed at intelligently and non-destructively ensuring the safety and reliability of everyday structures. Traditional SHM methods, which depend heavily on external sensors and monitoring devices, often incur high installation and maintenance costs, and can sometimes cause structural damage [1,2]. However, recent advancements in nanotechnology have given rise to self-sensing materials, a revolutionary approach to SHM that has garnered significant research interest. Among these, self-sensing cementitious composites stand out due to their unique properties and potential applications. Their distinguishing characteristic is their ability to monitor structural states non-intrusively [3,4]. Compared to traditional sensor installation methods, the application of these materials reduces the maintenance costs of monitoring systems, and improves the long-term reliability of the monitoring setup [5].

The core principle of self-sensing cementitious materials lies in the direct integration of sensing capabilities. This allows the sensors to autonomously monitor structural forces in real-time by observing changes in their own electrical resistance [6]. Currently, carbon and metal fillers are the two main types of conductive fillers in use. Sevim, O et al. noted an improved piezoresistive response in the sensor when 7.5% M graphene nanoparticles (GNPs) were added to the self-sensing cementitious material [7]. Suchorzewski, J et al. discovered that adding 0.05% MWCNT to concrete enabled damage detection at a tensile force of 85% Fmax, as evidenced by wedge splitting tests [8]. Roshan et al. developed self-sensing cementitious materials by integrating two types of conductive fillers, namely MWCNTs and GNPs. They uncovered a direct correlation between FCR changes and crack propagation within a structure [9]. In their study, Han et al. observed a direct link between the stability of piezoresistivity variations in self-sensing cementitious sensors and the applied external stresses [10]. Feng Xu’s experiments demonstrated that reactive powder cement concrete (RPC) with 1.0% nano-stainless steel powder (NSP) showed optimal strain-sensing sensitivity. Furthermore, the research depicted a cubic relationship, suggesting a decrease in the electrical resistance and drying shrinkage rate of RPC with the volume ratio of NSP [11].

Cyclic loading testing is a prevalent method for assessing the sensing capabilities of self-sensing cementitious materials [12,13]. By simulating the actual stress conditions experienced by sensors through repeated vertical loading and unloading, it can evaluate the sensing ability of self-sensing cementitious materials. This is significantly beneficial for assessing the stability and durability of sensors [14]. Saptarshi Sasmal and his colleagues found in their cyclic loading tests that the content of conductive fillers had a significant impact on the piezoresistive response. An ideal result was not achieved when the filler concentration was too high. Instead, the sensitivity of resistance changes under loading decreased. They had also discovered that the microstructure within the specimen changed during the loading process [15]. In the cyclic loading tests conducted by Yoo et al., they discovered that the self-sensing capacity of cement composites, inclusive of CF and GNF, under compression was not directly influenced by their conductivity. Furthermore, they identified that composites containing CNTs exhibited superior self-sensing capacity under cyclic compressive force at both 0.5 and 1 vol% [16].

In the study of self-sensing cementitious materials, fibers and powders emerge as the two primary categories of conductive fillers [17]. The hydrophobic nature of most fibrous fillers complicates their integration into these materials. In contrast, conductive powders, typically non-hydrophobic, disperse well in water [18,19,20]. Nanosilver, with its unique physical and chemical properties, has attracted significant interest in pressure-sensitive research. Unlike fibrous fillers, nanosilver’s small particle size, typically 10–50 nm, considerably reduces the composite system’s percolation threshold. Furthermore, nanosilver’s outstanding electrical properties offer substantial potential for advancements in the pressure sensitivity field [21,22]. Li et al. enhanced the surface conductivity of AgNW-PI films significantly by blending silver nanowires (AgNW) with polyimide (PI) polymers and employing a wet etching method for surface treatment. They further fabricated this material into a flexible pressure sensor. Experimental results revealed that this sensor demonstrated exceptional sensitivity, approximately 1.3294 kPa^−1^, under a pressure of about 600 Pa [23]. Alessandro Paghi et al. utilized control over the piezoresistive properties of the AgNP electrical network formed on PDMS foams to manufacture flexible and wearable pressure sensors. These sensors exhibit high deformation (GF) and pressure (S) sensitivities, allowing the detection of small displacements of up to 4 μm and low stresses of up to 25 Pa [24]. 

In the present study, a novel conductive filler, AgNP, was selected with the objective of addressing the challenges of high dosage, limited stability, and reversibility that are associated with traditional fillers. The stability and stress sensitivity of AgNP self-sensing cementitious materials were evaluated under two distinct loading regimes: long-cycle fixed amplitude intervals and long-cycle variable amplitudes.

## 2. Materials and Methods

### 2.1. Test Materials

**Figure 1:**
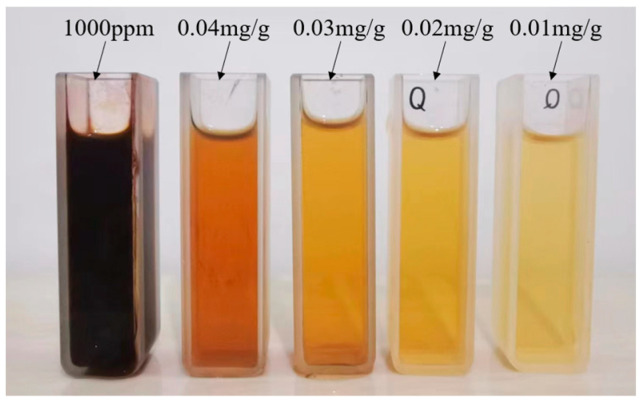
Aqueous solution of AgNPs.

**Figure 2:**
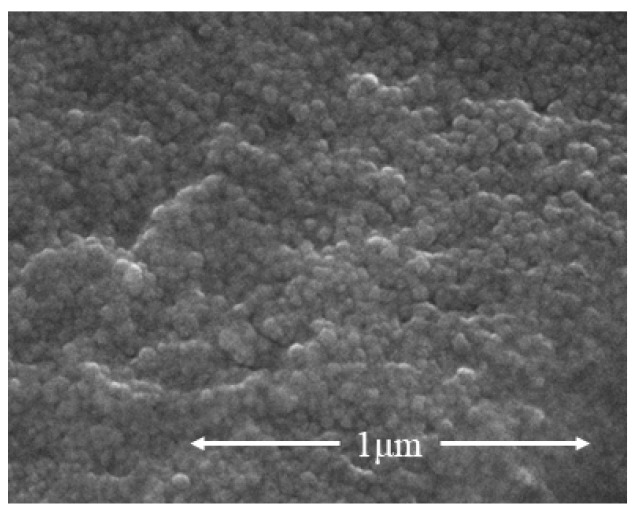
Scanning electron micrographs of AgNPs.

The experiment primarily utilized PO.42.5 silicate cement and a commercially provided aqueous solution of silver nanoparticles (AgNPs) with a concentration of 1000 ppm (refer to Figure 1). The AgNPs, as shown in the SEM photographs in Figure 2, had an approximate particle size of 10 nm. Six control groups were included in the experiment, all maintaining a consistent water-cement ratio of 0.4. The water content within the AgNPs, given their formulation as aqueous solutions, was incorporated into the specimen fabrication process. Table 1 outlines the exact ratios used.

### 2.2. Specimen Preparation

**Figure 3:**
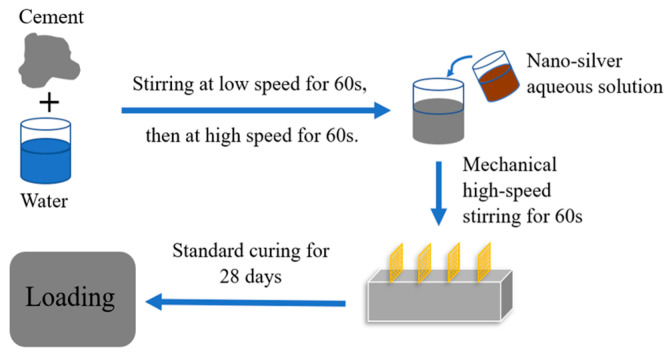
Flowchart of specimen preparation.

The specimens were prepared for by following the stipulations of GB/T 17671-2021 [25]. The specimens had external dimensions of 40 × 40 × 160 mm, with a water–cement ratio of 0.4 in the matrix phase. To avoid the measurement error caused by the coincidence of current and voltage poles, all specimens were made with a four-electrode design [26]. The resistance was measured with a Keysight Model 34461A digital multimeter, and the preparation process can be found in Figure 3.

**Figure 4:**
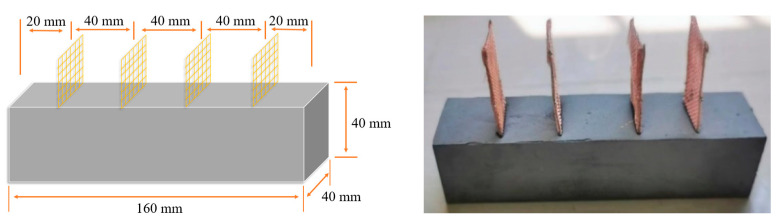
Photographs of specimen appearance.

Each electrode was composed of a 20-mesh copper mesh and a stainless-steel skeleton with a dimension of 25 mm × 75 mm. The electrodes were installed in a buried configuration. This design offers several benefits. It prevents the formation of a porous transition zone between the sheet electrode and the cementitious contact surface, ensuring strong contact. Additionally, it inhibits the formation of an adsorbed water layer that could otherwise compromise the mechanical properties of the specimen [27,28]. The electrode style is shown in Figure 4.

### 2.3. Test Methods and Evaluation Indicators

#### 2.3.1. Test Methods

**Figure 5:**
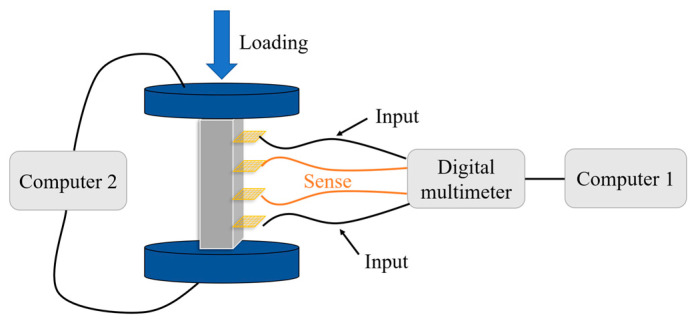
Flowchart of test loading for specimens.

In this study, the dispersion, percolation threshold, pressure-sensitive stability, and stress sensitivity of AgNPs were verified through absorbance tests, polarization tests, and cyclic loading tests, respectively. The loading principle is shown in Figure 5. 

In particular, the cyclic loading test was divided into two parts: equal amplitude cyclic loading under a long period and variable amplitude cyclic loading under a long period. For equal amplitude cyclic loading, a total of 120 cycles were applied, ranging from 1.25 MPa to 5 MPa, at a rate of 1 kN/s. In the case of variable amplitude cyclic loading, the amplitude gradually increased from 1.25 MPa to 3.4 MPa, 3.75 MPa, 4.7 MPa, and 6.25 MPa. The loading rate was 0.5 kN/s for cycles between 1.25 MPa and 3.4 MPa and 1 kN/s for the subsequent cycles.

#### 2.3.2. Analyzing Indicators

The relative rate of change of resistivity and the stress sensitivity factor are two important evaluation indexes for the self-perceiving cementitious pressure-sensitive properties.

The calculation of the resistivity formula (*ρ*) is shown as follows:

$$\rho =\frac{RS}{L}$$

where *R* is the measured resistance of the specimen, *S* is the cross-sectional area of the specimen, and *L* is the length between two neighboring electrodes.

Fractional change in the resistivity (FCR) is calculated as follows, where ρ is the value of the resistance at any moment in time, and $\rho_{0}$ is the calculated baseline resistance ($\rho_{0}$ was taken in this test to be the resistivity corresponding to 1.25 MPa in the last cycle).

$$FCR=\frac{\rho -\rho_{0}}{\rho_{0}}\times 100\%$$

The stress sensitivity factor (SS) characterizes the sensitivity of the self-perceiving cementitious material to the stress response.

$$SS=\frac{∆FCR\;[\%]}{∆\sigma \;[MPa]}$$

where ∆FCR is the relative change in FCR for a single cyclic loading process and ∆σ is the difference in stress during cyclic loading.

## 3. Test Results and Analysis

### 3.1. Absorbance of AgNPs

**Figure 6:**
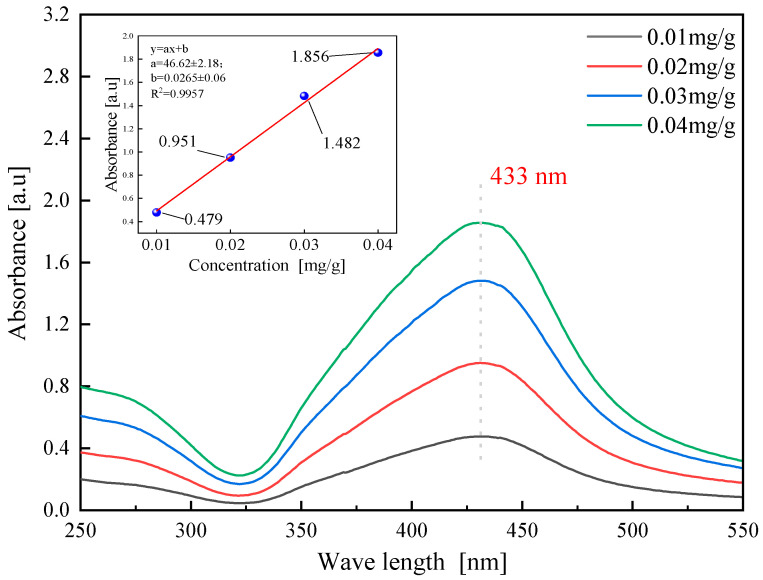
Absorbance data of AgNP aqueous solution at different concentrations.

If conductive fillers naturally agglomerate and are difficult to disperse in water, these agglomerates will adversely impact the pressure-sensitive properties of self-sensing cementitious materials. In this paper, the dispersibility of AgNPs in an aqueous solution was evaluated by UV spectrophotometer. According to the Beer–Lambert law (as shown in Equation (1)), the absorbance of the solution is directly proportional to the concentration. Consequently, the absorbance value of the solution serves as an evaluative indicator for filler dispersion in the liquid phase. A higher peak absorbance value demonstrates improved filler dispersion. For the absorbance test, the aqueous solution of AgNPs with a concentration of 1000 ppm needed to be diluted, as per Table 2. The specific test results are depicted in Figure 6. Equation (1) is as follows:

(1) $$A={\mathrm{lg}}^{\frac{1}{T}}=Kbc$$

where *A* is the absorbance and *T* is the transmittance ratio, which is the ratio of the outgoing light intensity (I) to the incident light intensity ($I_{0}$), *K* is the molar absorption coefficient, which is related to the nature of the absorbing substance and the wavelength λ of the incident light, *c* is the concentration of the absorbing substance in mol/L, and *b* is the thickness of the absorbing layer in cm.

**Figure 7:**
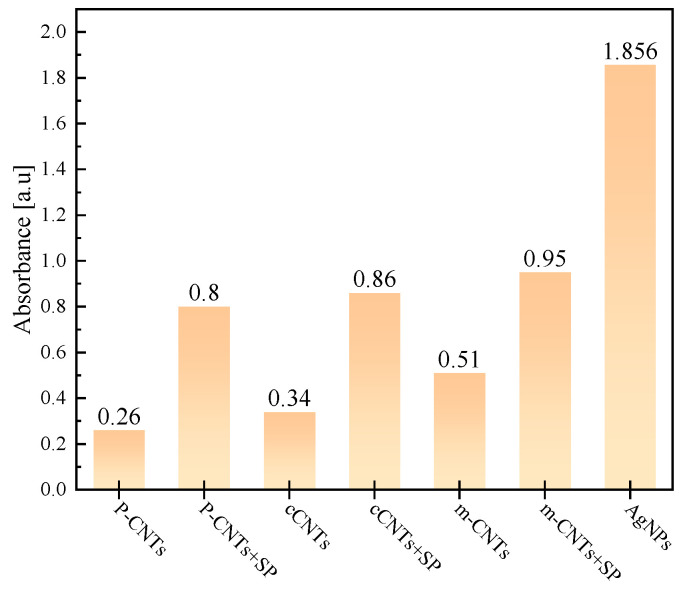
Comparison of absorbance data of AgNPs and CNTs.

As shown in Figure 6, the AgNP aqueous solution displays a significant absorption peak at a wavelength of 433 nm, with an absorbance of 1.856 at a concentration of 0.04 mg/g. The fitted peak value in the upper left corner of the graph further confirms that the absorbance data of AgNPs solutions at various concentrations comply with the Beer–Lambert law. According to research data by Sobolkina et al., the absorbance peak for CNTs falls between 0.5 and 1.2 at a wavelength of 260 nm [29]. Figure 7 compares the absorbance data of AgNPs with that of Li et al.’s study. The absorbance peaks for C-CNT and P-CNT materials, when dispersed with an SP dispersant, are around 0.8, which is lower than the 1.856 value of AgNPs [30]. This suggests that AgNPs demonstrate better dispersibility than traditional conductive fillers.

### 3.2. On Initial Resistance Affected by Water Content and Filler Dose

#### 3.2.1. Influence of Moisture Content

During the self-sensing cementitious application process, the specimen will interact with the surrounding environment. These actions will result in variations in the water content within the specimen and, consequently, will influence the specimen’s polarization effect. Therefore, in this section, the effect on water content was analyzed by controlling the drying time of water-saturated specimens.

Through specific tests, it was found that when the specimen was dried at 60 ℃ for 3 h, its quality no longer changed. Thus, it can be concluded that the specimen has attained a completely dry state at this point. Simultaneously, follow-up tests were conducted using 1-h intervals as test units, and the specimen was dried for three different durations: 1 h, 2 h, and 3 h. The moisture content was calculated using Equation (2), and the results are presented in Table 3 and Figure 7. Equation (2) is as follows:

(2) $$W=\frac{M_{0}-M_{n}}{M_{0}-M_{1}}\times 100$$

where $M_{0}$ is the mass of the specimen after water saturation, $M_{1}$ is the mass after complete drying, and $M_{n}$ is the mass of the specimen at different drying durations.

**Figure 8:**
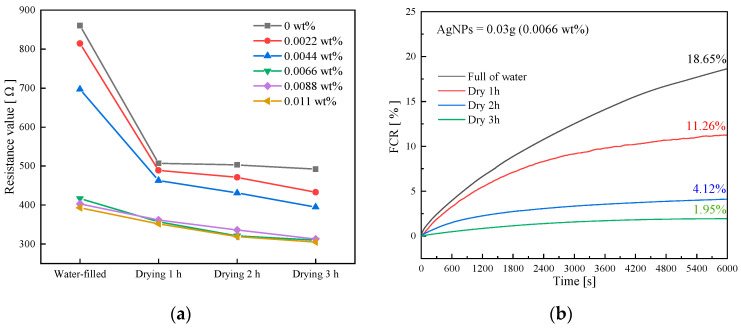
Resistance values and their variation with time. (**a**) Changes in initial resistance value vs. drying time. (**b**) Polarization curves of G3 at different drying times.

Figure 8a shows that the initial resistance value and moisture content of each specimen decreased the most after 1 h of drying, and then the trend of decreasing slowed down. This suggests a negative nonlinear correlation between the moisture content and the initial resistance of the specimen.

When the specimen’s water content exceeded 50%, most pores became saturated, making ionic conductivity the predominant factor. The conductive filler was enveloped by the liquid phase, hindering its contribution to conductivity due to short-circuiting. This elevated specimen resistance and reduced the sensor’s sensitivity to stress, diminishing the self-sensing capabilities [31]. When the moisture content fell below 30%, tunneling effects became prominent, diminishing the influence of ionic conductivity. A more consistent conductive pathway formed within the specimen [32,33].

Using the G3 specimen as an example, Figure 8b displays polarization data at varying water contents. The results show a gradual decrease in the growth rate of polarization curves as water content reduces. As drying time increases, FCR decreases from 18.65% to 1.95%. Previous discussions indicate that the reduction in polarization effect primarily results from ionic conductivity. However, as water content decreases, the polarization effect induced by ionic conductivity diminishes.

In summary, cyclic loading tests for self-sensing cementitious materials should be conducted at their natural moisture content, typically around 30%. This approach mitigates the adverse effects of ionic conductivity and provides a cushioning effect for cracks and fibers due to the presence of the liquid phase.

#### 3.2.2. Percolation Threshold

According to Ruschau et al., the trend of specimen resistivity does not show a linear correspondence with concentration [34]. The percolation threshold of different conductive fillers varies; it is closely related to the filler’s shape, conductivity, and size. Generally, the smaller the size of the filler, the lower the corresponding percolation threshold.

**Figure 9:**
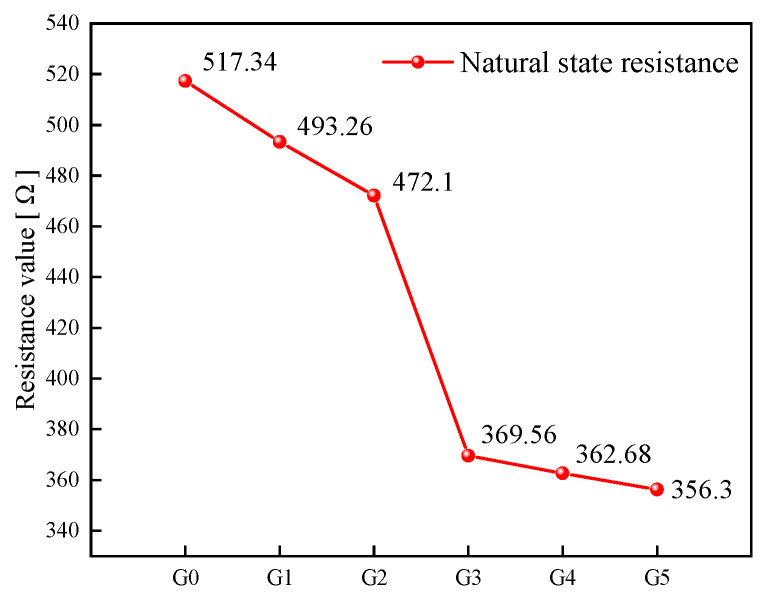
Correspondence between specimen resistance and doping amount of AgNPs.

Figure 9 displays the stabilized resistance value of self-sensing cementitious materials after polarization in its natural state. The figure shows that the resistance value of the specimen gradually decreases with increasing AgNP content and exhibits a sudden change at G3 (0.0066 wt%). Subsequently, the slope of the resistance value corresponding to the concentration slows down. Through extensive literature research, it can be confirmed that the AgNP content level of 0.0066 wt% in the cement reaches the percolation threshold. This indicates that self-sensing cementitious sensors in this state should yield the best pressure-sensitive test results.

### 3.3. Equal Amplitude Long-Cycle Loading

**Figure 10:**
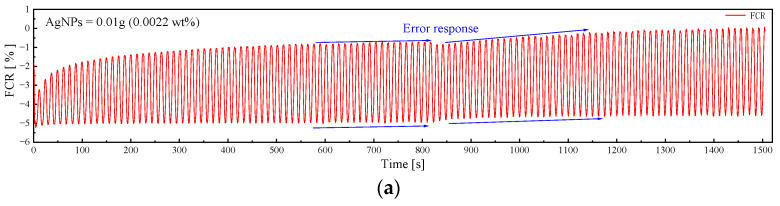

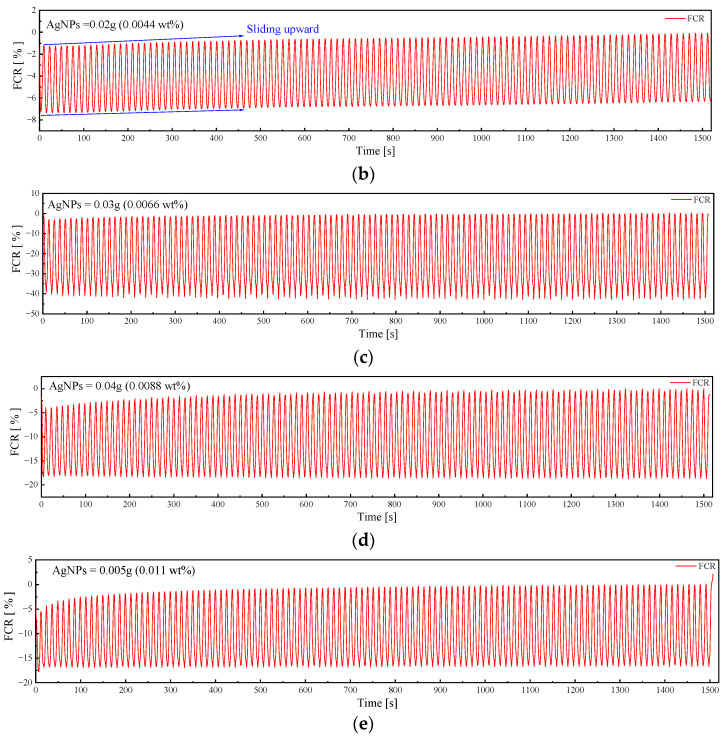
Cyclic loading test results of AgNP specimens. (**a**) G1. (**b**) G2. (**c**) G3. (**d**) G4. (**e**) G5.

Cyclic loading is the optimal test method for assessing the stability of the pressure-sensitive curve in the self-sensing cementitious materials. To accurately reflect the stability of AgNP self-sensing cementitious materials under the background of long cycle loading, the test involved 120 ultra-long cyclic loading cycles at a rate of 1 kN/s and stress variations ranging from 1.25 MPa to 5 MPa. Linear fitting was applied to supplement the evaluation of the self-sensing properties of the sensors. The results are shown in Figure 10.

#### 3.3.1. Loading Results

Figure 10 reveals that the poorest pressure sensitivity performances were observed in the G1 and G2 samples, while the G3, G4, and G5 samples demonstrate relatively robust pressure sensitivity stability. This aligns with earlier findings on percolation thresholds. It is worth mentioning that a substantial difference in pressure-sensitive performance exists between the G1 and G2 samples. The G1 sample showed error responses and late-stage curve up-slips. Conversely, G2 only experienced curve up-slips, with no issues in its pressure-sensitive response. 

**Figure 11:**
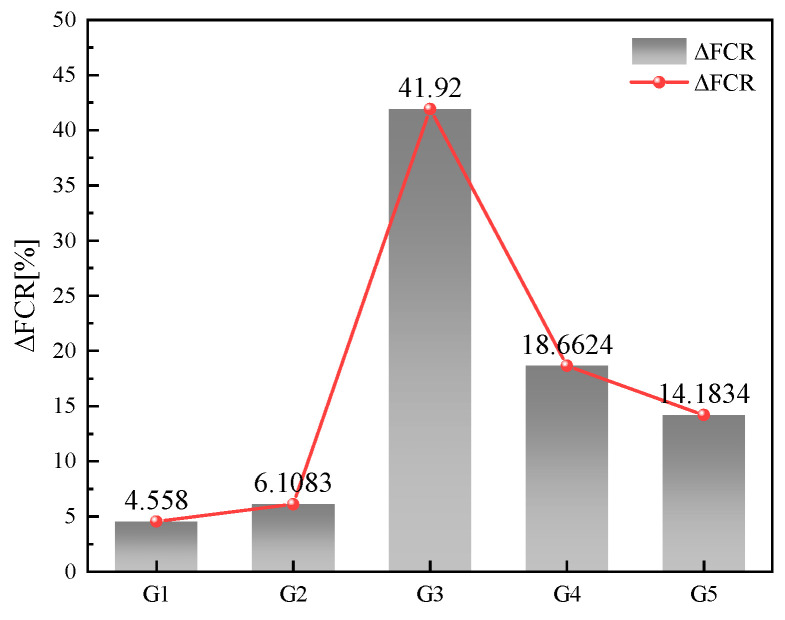
Variation in ΔFCR in AgNP specimens under loading.

ΔFCR represents the relative difference in FCR during each loading cycle, as shown in Figure 11. Upon observing the data, it is evident that ΔFCR exhibits a trend of initially increasing and then decreasing with the gradual rise in AgNP content. The peak value is observed at G3 (0.0066 wt%), reaching 41.92%. The data for AgNPs demonstrates a significant advantage when compared to other similar studies. In Mardani et al.’s study, the CNT self-sensing cementitious material showed a change in FCR of less than 10% over the loading interval of 0.625–6.25 MPa [35]. Similarly, in Suo et al.’s study on graphene oxide self-sensing cementitious material, the sensor’s FCR change was also less than 10% at pressures ranging from 0 to 15 MPa [36].

#### 3.3.2. Fitting Analysis of Loading Results 

**Figure 12:**
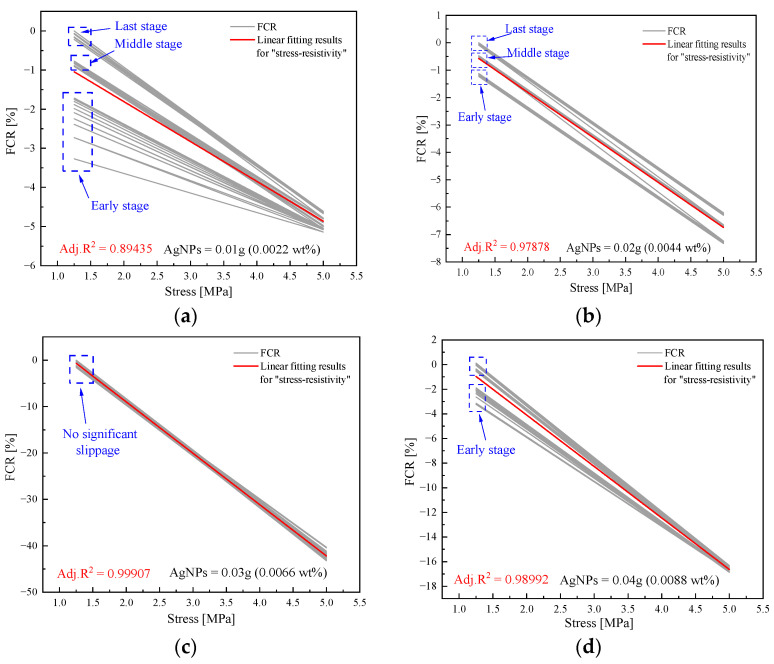

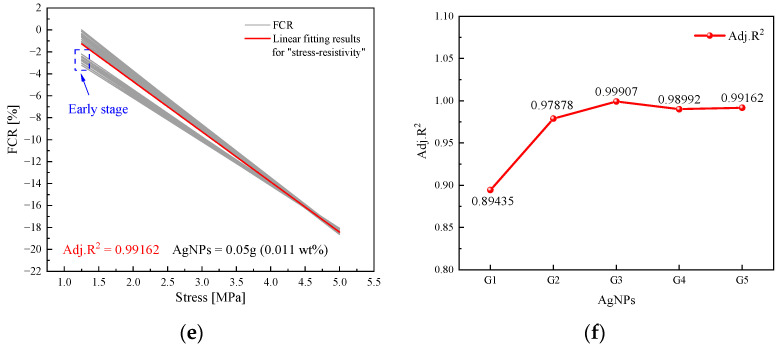
Results of the linear fitting of the “stress-resistivity” curve. (**a**) G1. (**b**) G2. (**c**) G3. (**d**) G4. (**e**) G5. (**f**) Adj.R^2^ of five groups.

To accurately assess the stability of sensor pressure sensitivity, an analysis was conducted on 30 cycles from the pre- (1st–10th), mid- (50th–60th), and post-phases (110th–120th). A linear fit was used to establish their “stress-resistivity” relationship. The goal of this fitting process is to obtain a continuous, sloping straight line without any discretization. The results are illustrated in Figure 12.

In Figure 12a, a noticeable dispersion is observed in the linear “stress-resistivity” curve for G1, particularly during the first 10 cycles (as indicated by the blue box in the figure). This observation suggests that the ΔFCR of the specimen gradually increases with the number of cycles. In Figure 12b, G2′s stress–resistivity curves during the initial, middle, and final stages do not exhibit any apparent discrete behavior; however, a significant translation phenomenon is observed. 

The fitting data for the adjusted R^2^ is shown in Figure 12f. Among all the specimens, G3 exhibits the least curve dispersion and curve translation, indicating the most stable response to cyclic loading. It achieves an R^2^ value of 0.99907, significantly higher than that of G1 and G2. While the R^2^ values for G4 and G5 are marginally lower than those of G3, the fitted resultant curves do not exhibit noticeable dispersion. This demonstrates that the stability of these two specimen groups is also relatively robust.

#### 3.3.3. Discussion

Based on the experimental results and the literature, the pressure-sensitive stabilization of AgNPs self-sensing cementitious materials is primarily influenced by three factors: the quantity of doped conductive filler, the polarization effect, and the elastic deformation of the cementitious matrix. 

According to Al-Dahawi, Luo et al. it was shown that the effect of the polarization effect is persistent and does not disappear during the loading process [37,38]. Consequently, an instable conductive network undermines the sensor’s capacity to counteract the polarization effect, leading to a slip in the pressure-sensitive curve. The data in Figure 10 and Figure 11 for G1 and G2 provide a detailed illustration of this phenomenon. Nevertheless, increased AgNP quantities can effectively control slippage. Nonetheless, excessive dosages do not ensure a positive outcome. The surplus use of conductive fillers can lead to AgNPs contacting each other, causing a shift from electron-leaping to contact conductivity. This shortens the sensor’s ΔFCR, as depicted in Figure 11.

In addition to the factors discussed earlier, elastic deformation of the cement matrix significantly influences pressure-sensitive stability. Continuous loading causes the matrix phase to compact, making recovery challenging without complete unloading. With more loading cycles, the variation in inter-particle spacing stabilizes. This phenomenon explains the reduced dispersion of the pressure-sensitive curve in Figure 12 with an increasing number of loading cycles.

### 3.4. Results and Analysis of Variable Amplitude Loading Tests

Variable amplitude loading can effectively demonstrate the stress sensitivity coefficient (SS) of self-sensing cementitious sensors, serving as a crucial reference for sensor applications. In this test, the amplitude varied over four intervals of 1.25 MPa to 3.4 MPa, 3.75 MPa, 4.7 MPa, and 6.25 MPa, none of them exceeded 50% of the ultimate compressive strength of the specimen.

#### 3.4.1. Loading Results

**Figure 13:**
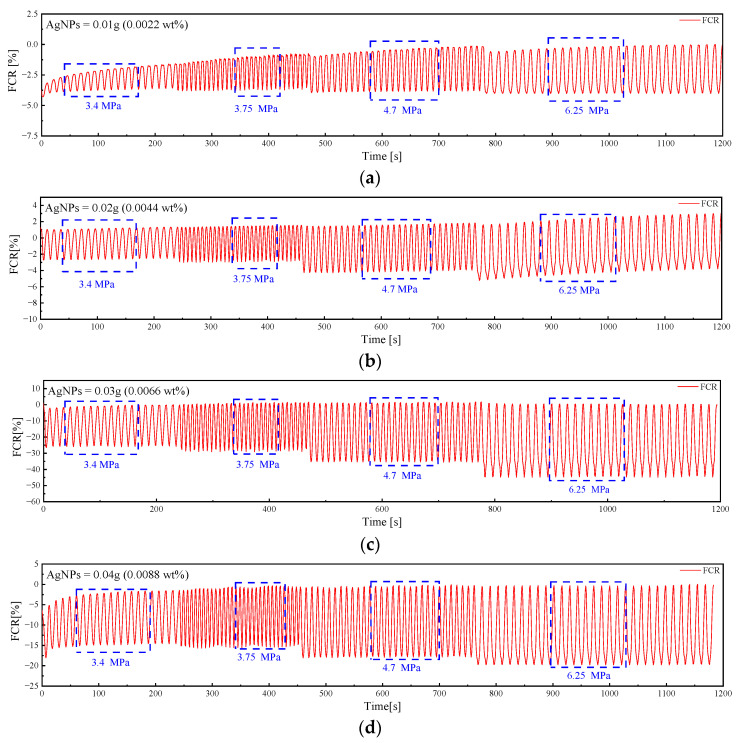

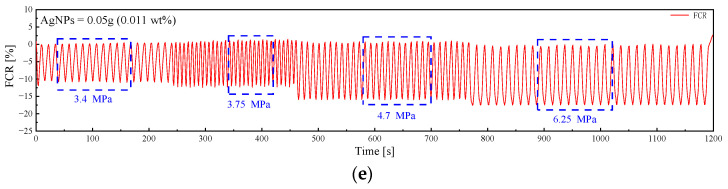
Variable amplitude loading test results for AgNP specimens. (**a**) G1. (**b**) G2. (**c**) G3. (**d**) G4. (**e**) G5.

Figure 13 depicts the pressure-sensitive response performance of five specimen groups subjected to varying loads. Among the test results, G1 exhibited one of the poorest outcomes, followed by G2. Within G1, the pressure-sensitive curve displayed instability, primarily attributed to the low AgNP content. Notably, G2 did not display a consistent upward sliding curve in response to low stresses. Instead, the curve shifted upward only under loads exceeding 4.7 MPa. In contrast, G3, G4, and G5 exhibited relatively improved pressure-sensitive performances due to increased AgNP doping.

**Figure 14:**
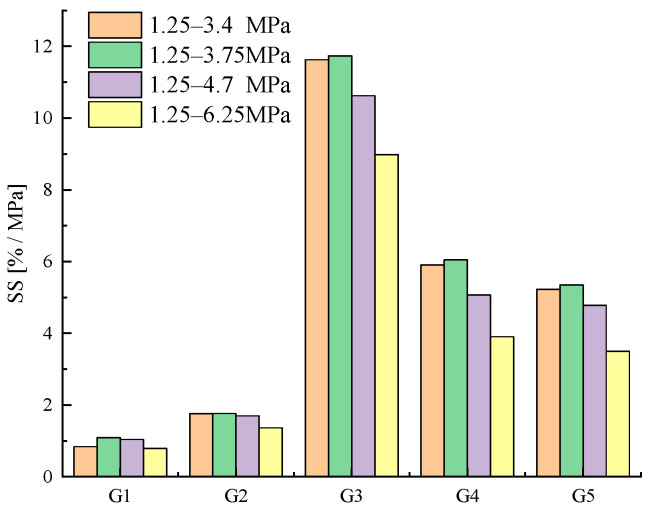
Variation in SS in AgNP specimens during variable amplitude loading tests.

**Figure 15:**
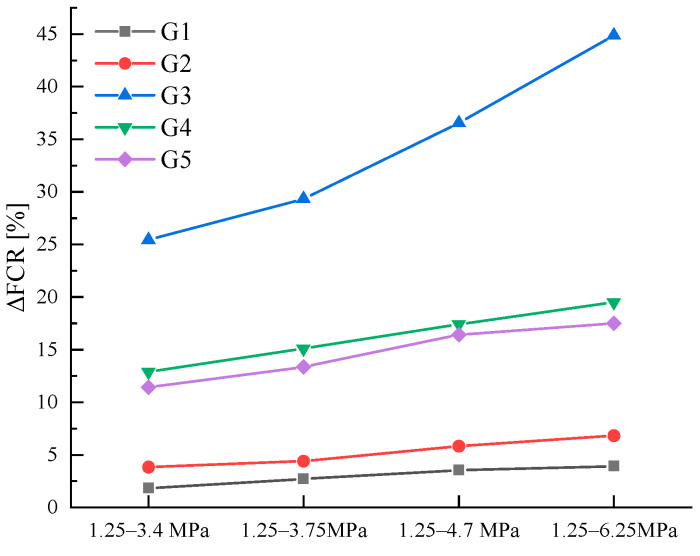
ΔFCR variation in AgNP specimens in variable amplitude loading tests.

Figure 14 and Table 4 provide a comparative analysis of the stress sensitivity factor (SS) for each specimen. The findings reveal a non-linear relationship between SS and ∆σ, wherein SS initially increases and subsequently decreases as ∆σ rises. Furthermore, it is evident from Figure 15 that although ΔFCR demonstrates a positive correlation with the external load, this correlation is not linear.

#### 3.4.2. Discussion

Experiments utilizing variable amplitude cyclic loading addressed two primary concerns: (1) the correlation between stress sensitivity (SS) and conductive filler doping, and (2) the variation in ∆σ and ∆FCR growth during incremental loading.

Following the percolation threshold theory, the specimen’s conductivity gradually transitions through the three regions of insulation, percolation, and conductivity as the conductive phase increases [33]. The SS in these three regions does not increase with doping; instead, it exhibits a peak response. High concentrations of conductive filler in the cementitious matrix increase particle contact, weakening the tunneling effect. This reduces the extent of ∆FCR change in the sensor, leading to decreased SS. Therefore, only sensors doped at the percolation threshold, where internal particles are uniformly dispersed, can simultaneously achieve peak SS and pressure-sensitive stability.

Yang et al. proposed that the nonlinear increase in ΔFCR with stress is attributed to changes in cracks within the specimen [39]. As stress increases, the crack width also expands, leading to physical isolation of the conductive path built by the conductive filler. Consequently, the tunnelling effect pathway becomes damaged, resulting in the nonproportional increase in ΔFCR. However, this viewpoint solely focuses on macro-level crack development and overlooks the dynamic changes in conductive pathways inside the specimen during stress variation. According to the tunneling effect principle, electron leaps occur only when particle spacing ranges from 1–10 nm. Nevertheless, under high stress, some particles compressed to 10 nm enable improved current flux by participating in tunneling, while others, originally capable of tunneling, become compressed to contact each other, losing the ability to facilitate electron jumps and transforming into contact conductivity. Therefore, the nonlinear growth in SS under loading is a consequence of a combination of these factors [40].

### 3.5. Analysis of the Application Effect of AgNPs

Tests in this paper reveal that a mere 0.03 g of AgNPs per 450 g of cement is needed for optimal results, amounting to just 0.0048 wt% of the total mass of the cementitious sensors. This amount is substantially lower than that of conventional conductive fillers. Table 5 shows that percolation thresholds of conventional self-sensing cementitious conductive fillers typically range within a few thousandths of a percent, contrasting significantly with AgNPs. The main reason for this variation is the difference in appearance and size of the conductive filler. As suggested by Qingzhong Xue, the percolation threshold of the composite system decreases with the reduction in the size of the conductive particles’ appearance [41]. This explains how AgNPs can reduce the amount of conductive filler by over 90% compared to conventional fillers while establishing more conductive pathways within the cementitious matrix.

**Figure 16:**
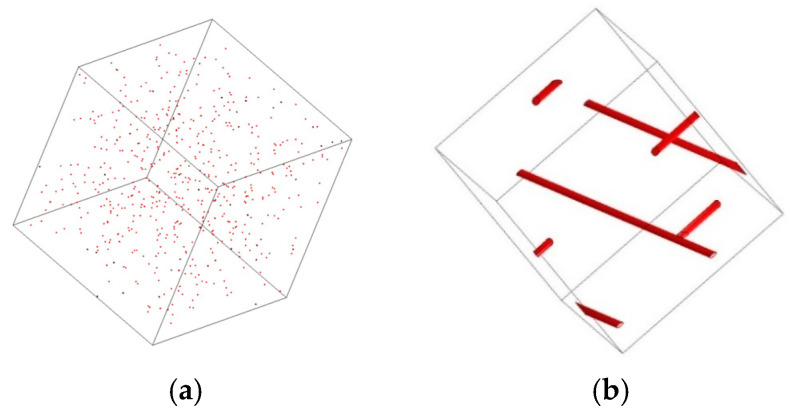
Comparison of dispersion effect of fillers, where (**a**) is AgNP and (**b**) is P-CNT.

**Figure 17:**
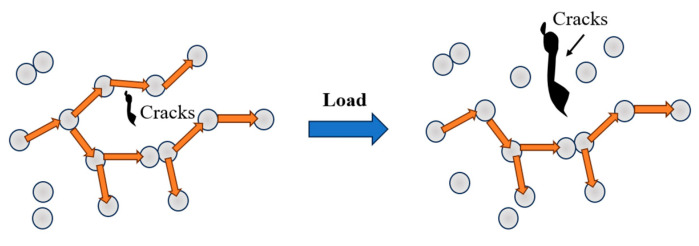
Diagram of the effect of cracks on the tunneling effect pathway.

Taking the P-CNT percolation threshold (0.03 wt%) from the group’s previous study as an example, the two materials were randomly dispersed using the Digimat analysis platform, and the dispersion parameters are presented in Table 6. Figure 16 illustrates the dispersion results. Despite AgNPs having only 1.59% of the mass of P-CNT, the number of fillers within the same computational cell is 1138 times greater than that of P-CNT. This observation indicates that AgNPs can establish a greater number of tunneling effect pathways inside the specimen, thereby ensuring the pressure-sensitive stability of the specimen. The specific principle is depicted in Figure 17.

## 4. Summary and Conclusions

In this study, five groups of self-sensing cementitious bases were prepared, each containing AgNPs at various doses: 0.0022 wt%, 0.0044 wt%, 0.0066 wt%, 0.0088 wt%, and 0.011 wt%. The dispersion, percolation threshold, polarization under varying water contents, pressure-sensitive stability, and stress sensitivity of AgNPs were investigated. The following conclusions were derived from the experiments:

1. At equivalent concentrations, AgNPs exhibited 1.15 to 9 times greater dispersibility in aqueous systems compared to conventional conductive fillers.
2. The percolation threshold of AgNPs in the cement matrix was determined to be 0.0066 wt% through polarization testing on five sets of specimens.
3. Long-term cyclic loading tests, both of equal and variable amplitude, revealed that self-sensing cementitious materials with 0.0066 wt% AgNPs exhibited optimal pressure-sensitive stability. The change in fractional change resistance (ΔFCR) reached up to 41.92%, and the maximum value of the stress sensitivity (SS) was as high as 11.736.
4. The utilization of AgNPs leads to a significant reduction in the quantity of conductive filler by approximately 90% or more. Their minute size and extremely high numbers contribute to a substantial increase in the number of conductive pathways within cementitious composite systems, thereby ensuring the stability of the pressure-sensitive effect.

## Figures and Tables

**Table 1:** Proportioning of AgNP specimens.

Group	Cement [g]	Water [g]	AgNPs Aqueous ^1^ [mL]	AgNPs [wt% of Cement]
G0	450	180	0	0
G1	450	170	10	0.0022
G2	450	160	20	0.0044
G3	450	150	30	0.0066
G4	450	140	40	0.0088
G5	450	130	50	0.011

^1^ There are 0.01 g of AgNPs per 10 mL of aqueous AgNP solution.

**Table 2:** Grouping of diluted solutions of AgNPs.

	Concentration	Aqueous AgNPs ^1^ [g]	Deionised Water [g]
Neutral solution	0.01 mg/g	1	99
	0.02 mg/g	2	98
	0.03 mg/g	3	97
	0.04 mg/g	4	96

^1^ The original solution concentration of the AgNP aqueous solution is 1000 ppm.

**Table 3:** Corresponding table of water content of specimens.

Group	Waterlogged State (*M*_0_)	Absolute Dry State (*M*_1_)	Moisture Content (After Drying) [%]		
			1 h	2 h	3 h
G0	502.2	491.3	55.96	24.77	0
G1	515.8	503.7	56.16	34.71	0
G2	509.4	497.6	55.93	28.81	0
G3	506.6	495.4	48.21	32.14	0
G4	509.1	495.9	54.39	31.06	0
G5	505.6	494.1	50.43	33.91	0

**Table 4:** SS and ΔFCR under different loads.

Stress/MPa	G1		G2		G3		G4		G5	
	ΔFCR ^1^	SS ^1^	ΔFCR	SS	ΔFCR	SS	ΔFCR	SS	ΔFCR	SS
1.25–3.4	1.834	0.838	3.853	1.761	25.436	11.628	12.916	5.904	11.431	5.225
1.25–3.75	2.714	1.086	4.411	1.764	29.339	11.736	15.115	6.046	13.359	5.344
1.25–4.7	3.555	1.034	5.828	1.695	36.534	10.628	17.414	5.066	16.417	4.776
1.25–6.25	3.931	0.786	6.815	1.363	44.879	8.976	19.528	3.906	17.489	3.498

^1^ The unit of ΔFCR is % and SS is (%/MPa).

**Table 5:** Electrical properties of cement-based sensors with different conductors.

Type of Matrix	Vol. or wt. Ratio in Cement [%]	ΔFCR	Reference
CNF	0.5 wt	0.9	Galao et al. [40]
	1.0 wt	1.0	
	2.0 wt	1.8	
CF	0.1 wt	13	Baeza et al. [42]
Nano-graphite	3.0 vol	2.5	Sun et al. [43]
	5.0 vol	15.6	

**Table 6:** Dispersion indicators for AgNPs and P-CNTs.

	Appearance	Dopant Amount ^1^	Volume Ratio	Calculate the Edge Lengths of the Microelements [nm]
P-CNT	Tubular	0.3 wt%	0.391%	1712
AgNPs	Spherical	0.0048 wt%	0.0117%	164

^1^ The dopant amount and volume ratio were calculated based on the total mass and volume of the cementitious sensors.

## Data Availability

Data are contained within the article.
